# Consumption of alcoholic beverages in Brazil: estimation of prevalence ratios – 2013 and 2019

**DOI:** 10.11606/s1518-8787.2023057004380

**Published:** 2023-03-29

**Authors:** Mariana Gonçalves de Freitas, Sheila Rizzato Stopa, Everton Nunes da Silva

**Affiliations:** I Universidade de Brasília Faculdade de Ciências da Saúde Programa de Pós Graduação em Saúde Coletiva Brasília DF Brasil Universidade de Brasília. Faculdade de Ciências da Saúde. Programa de Pós Graduação em Saúde Coletiva. Brasília, DF, Brasil; II Ministério da Saúde Secretaria de Vigilância em Saúde Departamento de Análise Epidemiológica e Vigilância de Doenças Não transmissíveis Brasília DF Brasil Ministério da Saúde. Secretaria de Vigilância em Saúde. Departamento de Análise Epidemiológica e Vigilância de Doenças Não transmissíveis. Brasília, DF, Brasil; III Ministério da Saúde Secretaria de Ciência, Tecnologia, Inovação e Insumos Estratégicos em Saúde Departamento de Ciência e Tecnologia Brasília DF Brasil Ministério da Saúde. Secretaria de Ciência, Tecnologia, Inovação e Insumos Estratégicos em Saúde. Departamento de Ciência e Tecnologia. Brasília, DF, Brasil; IV Universidade de Brasília Faculdade de Ceilândia Brasília DF Brasil Universidade de Brasília. Faculdade de Ceilândia. Curso de Saúde Coletiva. Brasília, DF, Brasil

**Keywords:** Alcohol Drinking, Risk Factors, Sociodemographic Factors, Gender and Health

## Abstract

**OBJECTIVES:**

To estimate the prevalence of weekly, monthly and abusive alcohol consumption in Brazil in 2013 and 2019, compare the period estimates, and verify the magnitude of the differences.

**METHODS:**

Analysis of data on alcohol consumption in the adult population (18 years or older) from the National Health Survey (PNS), 2013 and 2019. The number of interviewees in 2013 was 60,202 and 88,531 in 2019. The samples were characterized according to demographic, socioeconomic, health, and alcohol consumption variables and differences in proportions in the period were compared using Pearson’s c2 test, with Rao-Scott approximation and a 5% significance level. Multivariate Poisson regression models were estimated for the outcome variables of monthly, weekly and abusive consumption of alcoholic beverages, in order to estimate the magnitude of the differences between the 2013 and 2019 PNS estimates, using the prevalence ratio (PR). Models were adjusted per sex and age group and stratified per sex and demographic region.

**RESULTS:**

There was a difference in the distribution of the population according to race, occupation, income, age group, marital status, and education. There was an increase in alcohol consumption for all outcome variables, with the exception of weekly consumption in males. The PR of weekly consumption was 1.02 (95%CI 1.014–1.026), and in females the PR was 1.05 (95%CI 1.04–1.06). The highest PRs in the general population and per sex occur for abusive consumption. The increase in weekly consumption per region occurred in the South, Southeast, and Central-West regions.

**CONCLUSIONS:**

Males are the main alcohol consumers in Brazil; the PRs for both males and females show that there was an increase in monthly, weekly and abusive consumption in the research period; it is noteworthy that females have increased their consumption pattern with greater intensity than males.

## INTRODUCTION

The consumption of alcoholic beverages is present in different cultures and regions worldwide^1^and influenced by social, psychological, behavioral, economic, legal, and environmental factors^2^. These factors are expressed in the consumption pattern, in the practices carried out by the alcoholic beverage industry, and in each country’s public policies^2^. The volume of pure alcohol consumed and the consumption pattern (amount consumed and frequency) are factors directly related to the damage caused by the consumption of alcoholic beverages^3^. Alcohol is a relevant risk factor for morbidity and mortality in Brazil and the world and connected to more than 200 causes of death, including cancer, liver disease, circulatory system diseases, accidents, violence, and others^3^.

Data from the World Health Organization (WHO) indicate that the prevalence of alcohol consumption has increased in recent years^1^. In 2016, the prevalence of binge drinking (five or more drinks on a single occasion, in the last 30 days) among individuals aged 15 and over who consume alcoholic beverages was 39.5% worldwide and 40.5% % in the Americas region^1^. The total consumption of alcoholic beverages in the world population increased from 5.5 liters of pure alcohol per capita in 2005 to 6.4 liters in 2016, and could reach 7 liters in 2025. In the Americas, consumption in 2016 was 8 liters per capita and in Brazil it was 7.8 liters (13.4 in males and 2.4 in females), values higher than global estimates^1^.

Worldwide, alcohol consumption is associated with three million deaths and 131.4 million disability-adjusted life years (DALYs)^4^, corresponding, in 2016, to 5.3% of deaths and 5% of total DALYs. Considering the population aged between 20 and 39 years, 13.5% of deaths are associated with the consumption of alcoholic beverages^1^. A study carried out between 2013 and 2015 in 30 out of the 35 countries in the Americas, pointed out that more than 85,000 deaths (1.4% of the total) were totally attributable to alcohol, and 83.1% of those were in males^5^, with 64.9% of them occurring in individuals under 60 years of age^5^. Those deaths are preventable and relate to a high disease and premature mortality burden. Estimates indicate that the consumption of alcoholic beverages contributed to more than 300,000 deaths in the Americas in 2012 (5.5% of the total)^3^.

Data from the Global Burden of Disease 2017 indicate that 6.2% of deaths in Brazil are related to the consumption of alcoholic beverages, indicating that alcohol is the third risk factor for disease burden in Brazil and the fourth factor worldwide^6^. In addition to the morbidity and mortality burden, the consumption of alcoholic beverages also generates an important economic and social impact on the population.

Previous Brazilian studies on the consumption of alcoholic beverages and the related variables provide relevant information to understand the alcohol consumption in Brazil and who are the main consumers, according to demographic and socioeconomic characteristics^7 , 8^. In order to expand knowledge of the subject, this research adds information about patterns of consumption of alcoholic beverages and, mainly, about the intensity of increase in consumption in the Brazilian population during the period studied. This is a relevant point for public policy makers in dimensioning and facing the issues arising from alcohol use. The objectives of this study were to describe the Brazilian population’s profile according to socioeconomic and demographic characteristics, in 2013 and 2019; describe the prevalence of weekly, monthly and abusive alcohol consumption per sex; estimate the prevalence of weekly consumption of alcoholic beverages according to demographic region, and compare period estimates and verify the magnitude of differences.

## METHODS

This is a cross-sectional study on data from 2013 and 2019 National Health Survey (PNS) editions. The PNS is a population-based household survey conducted by the Brazilian Institute of Geography and Statistics (IBGE), in partnership with the Ministry of Health. Its data, collected from a representative sample, produced one of the most reliable portraits of the living and health conditions of the population residing in Brazil^9 , 10^.

The PNS sampling process was carried out by conglomerates in three stages of selection. In the first stage, the Primary Sampling Units (PSUs) were stratified, composed of the census tracts, described above, through simple random selection. In the second stage, households in each unit taken in the first stage were randomly selected. In the third stage, a resident was chosen with equiprobability among the other household residents. This resident was, at the time of the survey, 18 years old or older, in 2013, and 15 years old or older, in 2019.

The 2019 PNS questionnaire was prepared based on the previous edition, with the aim of maintaining the maximum comparison between editions, since the PNS is used to monitor national and international indicators, such as those of the 2011-2022^1^ Strategic Action Plan to Tackle Noncommunicable Diseases (NCDs) in Brazil^1^and the Sustainable Development Goals (SDGs)^12^.

The population included in the survey resides permanently in private households in Brazil, that is, special census tracts such as military bases, penitentiaries, long-stay institutions, convents, hospitals, and sectors located in indigenous lands are not included in the sample.

The 2013 sample consisted of 69,994 households, resulting in 64,348 interviews, with an 8.1% non-response rate. In 2019, 108,525 households were visited and 94,114 interviews were conducted, with a 6.4% non-response rate. Due to its sampling process, it is necessary to use an algorithm capable of considering the effects of its stratification and conglomeration to estimate indicators. Thus, weighting factors were calculated by the inverse of the selection probability at each stage, adding a correction factor for losses^13^.

This study analyzed data pertaining to the adult population (18 years of age or older), from both PNS editions, referring to the consumption of alcoholic beverages reported by the interviewees. The number of respondents aged 18 or over in 2013 was 60,202 and 88,531 in 2019.

The outcomes of interest were considered: monthly consumption of alcoholic beverages, obtained through the question P27 “How often do you consume alcoholic beverages?”; weekly alcohol consumption, obtained through question P28 from the PNS 2013 “How many days a week do you usually drink alcohol?” and P28a, from the PNS 2019, “How many days a week do you usually consume an alcoholic beverage?”; and abusive consumption of alcohol in the last 30 days, obtained from question P32, from the PNS 2013, “In the last 30 days, have you consumed five or more alcoholic drinks on a single occasion?” (if male), or “In the last 30 days, have you consumed four or more alcoholic drinks on a single occasion? (if female), and question P32a from the 2019 PNS “In the last 30 days, have you consumed five or more alcoholic drinks on a single occasion?”. For clarification purposes, a dose of alcoholic drink is equivalent to: one can of beer or one glass of wine or one shot of *cachaça* , whiskey or any other distilled alcoholic beverage. We emphasize that there was a change in the classification of abusive consumption for females adopted by the 2019 PNS, in accordance with WHO guidance^1^.

For the calculation of weekly consumption and abusive consumption, all questionnaire interviewees over 18 years of age were considered in the denominator. The variable *daily consumption* was created from questions P028 and P029 by multiplying these questions and dividing by seven, to estimate the daily dose of alcohol consumed. This value was then multiplied by 12 to estimate the daily dose in grams. For this variable, the dose standard of 12 pure alcohol grams per dose was used.

The samples in both years of study were characterized according to demographic and socioeconomic variables: sex (female or male); age group (18 to 29, 30 to 39, 40 to 59, 60 years and over); race/color (white, black and brown); education levels (no schooling up to incomplete elementary school, complete elementary school up to incomplete secondary school, complete high school up to incomplete higher education, complete higher education); marital status (married, divorced or separated, widow/widower and single); occupation (employed or unemployed); income in minimum wages (MW) (no income, up to ½ MW, from ½ MW to 1 MW, from 1 MW to 2 MW, greater than 2 MW ); Major Regions (North, Northeast, South, Southeast, Central-West), and census sector (urban or rural). The sample was also characterized by health variables and variables related to the consumption of alcoholic beverages: self-perceived health (very good/good, fair, poor/very poor); tobacco consumption (yes, no); consumption of alcoholic beverages per weekdays (0, 1, 2, 3-6, 7); consumption of alcoholic beverages in doses (1, 2-4, 5-9, > 10), and consumption of alcoholic beverages in grams per day (12, 24, 36, 48, 60 and 72).

Analyzes were performed on a single database, where the aforementioned variables were grouped to enable comparison between the two PNS editions. The comparison was made by estimating the proportions and their 95% confidence intervals. With the objective of verifying possible changes between data from the two PNS editions, Pearson’s c2 test was used, with Rao-Scott approximation and a 5% significance level. Multivariate Poisson regression models were estimated considering the outcome variables *monthly consumption of alcoholic beverages, weekly consumption of alcoholic beverages* and *abusive consumption of alcoholic beverages* , in order to estimate the magnitude of the differences between 2013 and 2019 PNS estimates, by means of the prevalence ratio (PR). The models were adjusted per sex and age group. The models that were stratified per sex were adjusted per age group. Analyzes were carried out on Stata 14.0 software, using the survey module, which considers the effects of complex sampling in parameter estimation.

Both PNS editions were approved by the National Research Ethics Committee (CONEP), under opinion No. 328.159, of June 26, 2013, and No. 3.529.376, of August 23, 2019. PNS data are public and available at https://www.pns.icict.fiocruz.br/bases-de-dados/^14^.

## RESULTS

Table 1 presents the Brazilian population’s profile according to socioeconomic and demographic characteristics in 2013 and 2019. We observed an increase in the self-report of black people and a decrease of white people between 2013 and 2019 (p<0.001); an increase in the proportion of older population (60 years and over), from 18.06% to 21.61%, and a decrease in the younger population aged 18 to 29, from 26.11% to 22.10% ( p < 0.001); an increase in the proportion of divorced or legally separated people, from 6.41% to 7.08%; an increase in the proportion of individuals with higher education (from 12.72% to 15.83%) and a reduction in the population without schooling and with incomplete elementary school (from 39.98% to 34.76%) (p<0.001); a reduction in the employed population, from 94.72% to 92.78% (p < 0.001); an increase in the proportion of people earning half the minimum wage, from 19.40% in 2013 to 21.27% in 2019, and a reduction in the number of people earning more than two minimum wages, from 21.47% to 20, 62% (p = 0.001). There was no statistical difference between years in relation to the proportions of sex, demographic region, and census sector.

**Table 1:** Percentage distribution of the Brazilian population aged 18 years and over, according to demographic and socioeconomic characteristics and comparison of differences in the period, Brazil 2013 and 2019.

	2013		2019		p^a^
	
	%	95%CI	%	95%CI	
Sex					
Female	52.90	52.13–53.66	53.16	52.56–53.76	
Male	47.10	46.34–47.87	46.84	46.24–47.44	0.5922
Age group					
60 +	18.06	17.48–18.65	21.61	21.08–22.16	< 0.001
40 to 59 years old	34.24	33.59–34.91	35.30	34.71–35.89	
30 to 39 years old	21.59	21.0–22.19	20.99	20.48–21.51	
18 to 29 years old	26.11	25.48–26.76	22.10	21.51–22.70	
Race					
White	48.21	47.39–49.03	43.91	43.18–44.64	< 0.001
Black	9.26	8.81–9.73	11.64	11.23–12.06	
Brown	42.53	41.75–43.31	44.45	43.77–45.14	
Schooling					
No education to incomplete primary education	38.98	38.12–39.84	34.76	34.1–35.42	< 0.001
Complete primary education to incomplete secondary education	15.52	14.98–16.07	14.48	14.06–14.91	
Complete secondary education to incomplete higher education	32.79	32.08–33.5	34.94	34.33–35.55	
Complete higher education	12.72	12.02–13.45	15.83	15.19–16.48	
Marital status					
Married	44.46	43.67–45.25	43.88	43.2–44.55	0.0285
Divorced or legally separated	6.41	6.09–6.76	7.08	6.82–7.36	
Widower/ widow	6.66	6.36–6.98	6.85	6.58–7.14	
Single	42.47	41.73–43.22	42.19	41.52–42.85	
Occupation					
Employed	94.72	94.28–95.13	92.08	91.62–92.52	< 0.001
Unemployed	5.28	4.87–5.72	7.92	7.48–8.38	
Income					
No income	1.02	0.89–1.17	0.89	0.80–0.98	0.0011
½ minimum wage	19.40	18.81–20.00	21.27	20.72–21.82	
1 minimum wage	29.32	28.57–30.08	29.08	28.47–29.69	
2 minimum wages	28.79	28.11–29.49	28.16	27.55–28.77	
>2 minimum wages	21.47	20.61–22.36	20.62	19.89–21.36	
Demographic region					
North	7.47	7.25–7.70	7.85	7.59–8.12	0.3862
Northeast	26.46	25.92–27.01	26.45	25.92–27.00	
Southeast	43.91	43.22–44.61	43.44	42.65–44.24	
South	14.75	14.33–15.18	14.68	14.27–15.11	
Central-West	7.41	7.21–7.62	7.57	7.293–7.854	
Census sector					
Rural	13.83	13.37–14.30	13.82	13.44–14.22	0.9900
Urban	86.17	85.70–86.63	86.18	85.78–86.56	

Source: National Health Survey, 2013 and 2019

95%CI: 95% confidence interval.

^a^ Pearson’s c2 with Rao-Scott approximation.

Table 2 presents variables related to health and alcohol consumption. There was no statistical difference between years in relation to self-rated health. There was a reduction in the prevalence of smoking in the period, from 14.65% to 12.59%, with p value < 0.001. The variables related to the consumption of alcoholic beverages presented changes in the proportions: number of weekdays that the person consumes alcohol; number of doses consumed, and daily consumption of alcohol in grams. It is observed that there was an increase in consumption for these three variables between 2013 and 2019 (p<0.001). In 2013, 13.55% of the population reported consuming an average dose of alcohol per day, and in 2019 this proportion increased to 14.70%. Regarding the interviewees, 5.41% reported consuming 24 grams of alcohol per day in 2013, rising to 6.06% in 2019. In 2013, 2.40% reported consuming 36 grams of alcohol per day, while in 2019 the percentage rose to 2.83% (p<0.001).

**Table 2:** Percentage distribution of the Brazilian population aged 18 years and over, according to health alcoholic beverage consumption characteristics and comparison of differences in the period, Brazil 2013 and 2019.

	2013		2019		p^a^
	
	%	95%CI	%	95%CI	
Health self-perception					
Very good/good	66.19	65.48–66.90	66.11	65.5–66.72	0.9752
Fair	28.01	27.37–28.67	28.10	27.57–28.64	
Poor/very poor	5.79	5.49–6.11	5.79	5.53–6.05	
Smoker					
No	85.35	84.85–85.84	87.41	87.01–87.81	< 0.001
Yes	14.65	14.16–15.15	12.59	12.19–12.99	
Number of weekdays					
0	9.72	8.91–10.59	12.09	11.39–12.83	0.0001
1	40.91	39.51–42.33	39.28	38.20–40.36	
2	27.09	25.80–28.42	26.95	25.98–27.94	
3 to 6	15.20	14.25–16.21	15.76	14.94–16.61	
7	7.08	6.40–7.83	5.93	5.41–6.49	
Number of drinks					
1	18.86	17.64–20.14	21.13	20.32–21.97	< 0.001
2 to 4	44.30	42.84–45.77	45.85	44.81–46.90	
5 to 9	22.48	21.32–23.69	21.21	20.41–22.04	
>10	14.36	13.48–15.28	11.81	11.23–12.40	
Consumption of alcoholic beverages in grams per day					
0	76.08	75.38–76.77	73.61	73.03–74.19	< 0.001
12	13.55	13.00–14.11	14.70	14.25–15.16	
24	5.41	5.08–5.77	6.06	5.79–6.34	
36	2.40	2.18–2.64	2.83	2.58–3.11	
48	0.84	0.71–0.99	1.05	0.93–1.18	
60	0.49	0.42–0.58	0.57	0.49–0.66	
72	1.24	1.11–1.38	1.18	1.05–1.33	

Source: National Health Survey, 2013 and 2019

95%CI: 95% confidence interval

^a^ Pearson’s c2 with Rao-Scott approximation.

Table 3 presents the monthly, weekly, and abusive consumption of alcoholic beverages in 2013 and 2019. For all alcohol consumption variables, there was an increase in consumption with a statistical difference between years, with the exception of weekly consumption in males.

**Table 3:** Prevalence and adjusted prevalence ratios of monthly, weekly and abusive consumption of alcoholic beverages in the Brazilian population aged 18 years and over, Brazil, 2013 and 2019.

	2013		2019		PR^a^	95%CI	p^b^
	
	%	95%CI	%	95%CI			
Monthly consumption of alcoholic beverages	26.49	25.76–27.24	30.02	29.41–30.64	1.009	1.007–1.011	< 0.001
Monthly consumption of alcoholic beverages - males	39.23	38.14–40.34	41.16	40.28–42.05	1.004	1.002–1.007	0.001
Monthly consumption of alcoholic beverages - females	15.14	14.38–15.94	20.20	19.48–20.94	1.014	1.011–1.017	< 0.001
Weekly consumption of alcoholic beverages	23.92	23.23–24.62	26.39	25.81–26.97	1.020	1.014–1.026	< 0.001
Weekly consumption of alcoholic beverages - males	36.27	35.20–37.34	37.09	36.20–37.99	1.006	0.999–1.012	0.078
Weekly consumption of alcoholic beverages - females	12.92	12.23–13.65	16.96	16.29–17.64	1.053	1.041–1.065	< 0.001
Alcohol abuse	13.62	13.11–14.15	17.06	16.59–17.55	1.045	1.038–1.053	< 0.001
Alcohol abuse - males	21.52	20.62–22.45	26.00	25.17–26.84	1.037	1.028–1.046	< 0.001
Alcohol abuse - females	6.59	6.14–7.07	9.20	8.74–9.67	1.069	1.052–1.083	< 0.001

Source: National Health Survey, 2013 and 2019

95%CI: 95% confidence interval

^a ^PR: adjusted per sex and age

^b^ Pearson’s c2 with Rao-Scott approximation.

The prevalence ratio (PR) data for 2019 compared to 2013 show that, in the general population, there was an increase in monthly consumption of alcoholic beverages in the period (PR of 1.009 CI95% 1.007–1.011). Monthly alcohol consumption among males had a PR of 1.004 (95%CI 1.002–1.007), and among females the PR was 1.014 (95%CI 1.011–1.017), demonstrating that the greatest increase in consumption occurred in females ( Table 3 ).

Data on weekly consumption of alcoholic beverages also reveal an increase in consumption in the period (PR 1.02 CI95% 1.014–1.026), and in females the PR was 1.05 (CI95% 1.04–1.06). The highest PRs in the general population and per sex occur for abusive consumption. It is observed that males are the main alcohol consumers in Brazil, and the PRs for both males and females show that there was an increase in both monthly and weekly consumption and also in abusive consumption in the period. It is noteworthy that there was an increase in the pattern of consumption with greater intensity among females compared to males ( Table 3 ).

It is observed that the prevalence of weekly consumption of alcoholic beverages in Brazil differs between demographic regions ( Table 4 ). In 2013, the South region presented the highest consumption, followed by Central-West, Southeast, Northeast, and North regions. This order changes in 2019, when the Southeast region occupies the second place in terms of prevalence of weekly consumption of alcoholic beverages in Brazil and the Central-West region ranks third. The Southeast region presented the greatest increase in weekly alcohol consumption in the country (RP=1.038 95%CI 1.028–1.049), followed by the South (RP=1.22 95%CI 1.008–1.036) and Central-West (PR = 1.016; CI95% 1.004 to 1.029) regions. The North and Northeast regions presented no statistical difference for the PR.

**Table 4:** Prevalence and adjusted prevalence ratios of weekly consumption of alcoholic beverages in the Brazilian population aged 18 years and over, according to demographic region, Brazil, 2013 and 2019.

	2013		2019		PR^a^	95%CI	p^b^
	
	%	95%CI	%	95%CI			
North	18.80	17.40–20.29	17.21	16.29–18.17	0.991	0.976–1.006	0.239
Northeast	22.28	21.26–23.34	20.72	19.96–21.50	0.993	0.984–1.002	0.148
Southeast	24.09	22.85–25.38	29.71	28.62–30.83	1.038	1.028–1.049	< 0.001
South	28.18	26.33–30.12	31.21	29.79–32.68	1.022	1.008–1.036	0.001
Central-West	25.38	23.89–26.94	27.28	25.97–28.64	1.016	1.004–1.029	0.011

Source: National Health Survey, 2013 and 2019

95%CI: 95% confidence interval

^a ^PR: adjusted per sex and age

^b^ Pearson’s c2 with Rao-Scott approximation.

## DISCUSSION

The PRs for the period show that there was an increase in monthly, weekly and abusive consumption of alcoholic beverages between 2013 and 2019, which was also observed in the analysis stratified by sex. For all indicators of alcohol consumption pattern analyzed in this study, the increase in prevalence measured by the PR in the period was higher in females compared to males. Although the consumption of alcoholic beverages among females has increased more significantly, males remain the main consumers of alcoholic beverages, which is in line with international estimates^1^.

The increase in weekly consumption of alcoholic beverages was also observed in the country’s Southeast, South and Central-West regions, which already had the highest prevalence of consumption. The lowest consumption occurred in the North and Northeast regions. The WHO data indicate that the highest prevalence of alcohol consumption occurs in countries with higher income^1^. This may help understand why the lowest prevalence were observed in the North and Northeast regions. Considering the prevalence of abusive consumption in the capitals, the highest prevalence occurs in the cities of Salvador and Florianópolis, while the lowest consumption of alcohol occurs in the cities of Manaus and Fortaleza. Abusive consumption is higher among males between 25 and 34 years of age and with high levels of education^15^.

A study with data from the 2013 PNS shows that recent consumption (in the last 30 days) of alcohol in that year was 26.5%^7^. In our study, these results correspond to monthly alcohol consumption. White, younger, single and urban males presented association with recent alcohol consumption. In females, this consumption was associated with those belonging to a younger age group, with higher education, single or separated, and living in an urban area. These data add information to our study, since its main objective was to investigate the differences in the population profile between 2013 and 2019, as well as the evolution of the prevalence of alcohol consumption in the period.

Research that investigated the heavy consumption of alcoholic beverages in Brazil based on data from the 2013 and 2019 PNS identified that 6.1% of Brazilians had a pattern of heavy consumption in 2013, increasing to 7.3% in 2019. The highest prevalence was in single young males with low education, and living in urban areas. The aforementioned results differ from data on episodic heavy drinking in this analysis because the Center for Disease Control and Prevention (CDC) reference for heavy drinking was used, which is defined as the intake of eight or more drinks per week for females and 15 or more doses for males^16^. The standard referred to by the CDC implies a greater number of doses and considers consumption throughout the week; however, in this study, abusive consumption on the same occasion was considered.

Heavy episodic consumption increases the risk of accidents and violence, as well as the development of alcohol-related diseases. A literature review pointed to the need for additional studies that investigate the role of the relationship between the adopted consumption pattern and the average volume of alcohol consumed, in order to obtain more accurate risk estimates and better understand the nature of alcohol-disease relationships^17^.

The increase in alcohol consumption in the Brazilian population follows the upward trend in the Americas predicted by the WHO^1^. This intensification of consumption is a wake-up call for public policy makers. Several studies currently reveal that there is no consumption of alcoholic beverages that does not pose health risks^18 , 19^. These studies even claim that the possible cardiovascular protective effects are lower than the damage related to the consumption of alcoholic beverages^19^. Weekly alcohol consumption was positively associated with premature mortality, and the main causes of excessive mortality were cancer, vascular diseases, and external causes^20^. Considering the increase in alcohol abuse, it should be noted that the risk of all-cause mortality increases with the increase in the amount of alcohol consumed^19 , 20^.

During the Covid-19 pandemic, there was a change in the pattern of alcohol consumption, associated with social isolation, age group, and mental health^21^. Research into the association between increased alcohol consumption and mental health identified a PR of 1.64 (95%CI 1.21–2.23) among individuals with depressive symptoms compared to individuals without these symptoms, and a PR of 1.41 (95%CI 1.20–1.66) in people with anxiety symptoms^21^. The association between alcohol and mental health was more expressive among people over 60 years old^21^. Data from a cross-sectional study show that 17.6% of the Brazilian population reported increasing alcohol consumption during the pandemic, and among individuals aged 30 to 39 years this increase was of 24.6%. There was no difference in the increase in consumption between sexes^22^. Further studies will be needed to find out whether this increase may represent a new consumption pattern, which may be identified in future PNS editions.

Considering the policies and guidelines that direct the actions of countries in dealing with issues arising from the consumption of alcoholic beverages, the Global Strategy to Reduce the Harmful Use of Alcohol^23^ stands out, which defines actions that regulate the commercialization of alcohol, availability, and taxation, among other measures. In 2018, the WHO launched the Safer technical package, which represents a strengthening and updating of the Global Strategy and includes five areas of national and subnational intervention^24^. Safer is a tool that helps countries to achieve the goals defined in the Sustainable Development Goals^25^. Monitoring of alcohol consumption in liters of pure alcohol per capita in individuals aged 15 and over is of the SDG targets ^25^.

In Brazil, the 2011-2022 Strategic Action Plan to Tackle Chronic Noncommunicable Diseases defined as national goals the reduction of the prevalence of alcohol abuse by 10%, and as strategies the increase in taxes on alcohol, measures of inspection of sale of alcoholic beverages to people under 18 years of age, control of sale points, and educational measures^11^. In 2021, the new edition of the plan was published, for the period from 2021 to 2030, which brought the balance of the first decade. Vigitel 2019 data indicate that abusive consumption in Brazil was 18.8%^26^. The action plan goal was to reduce abusive consumption to a prevalence of 16.3% in 2022. Thus, projections by the Ministry of Health indicate that Brazil would not reach this goal within the period of the first plan^27^. Therefore, the target was renewed in the 2021–2030 plan. The increase in price and decrease in availability are cost-effective and are among the defined best practices^23 , 28^. Alcohol taxation is also an effective mechanism, especially among adolescents and individuals who present heavy consumption of alcoholic beverages^29^.

In Brazil, the Alliance for Tobacco Control (ACT) does advocacy work with legislators to strengthen the alcohol regulatory agenda. The Public Ministry of São Paulo is responsible for the campaign: “Beer is also alcohol”^30^. Such campaign proposes the amendment of article 1 of Federal Law No. 9.294/9631, which allows beer advertising, as the drink has an alcoholic strength of less than 0.5 degrees Gay-Lussac. Alcohol advertising campaigns have a strong influence on the public that does not consume yet, including teenagers, a group of potential consumers of interest to the industry^2 , 29^. The alcoholic beverage industry plays an important role in the national and international scene related to consumption, since it influences policy formulation, managing the commercial interests of industries in the space of political decisions^32 , 33^.

## CONCLUSION

The change in the alcohol abuse indicator in the 2019 PNS compared to the way this data was captured in 2013 can be mentioned as a study limitation. In 2019, the survey considered alcohol abuse the intake of five drinks on the same occasion for males and females^10^. In 2013, in turn, the survey considered alcohol abuse the intake of four drinks for females and five drinks for males on a single occasion^9^. Thus, the increase in the abusive consumption of alcoholic beverages among females between 2013 and 2019 could be even more significant, since the change in the aforementioned indicator implied an increase in the number of doses consumed by them. This change came from the Ministry of Health team to align with WHO recommendations^1^. In addition, in 2013 this question about alcohol abuse was answered by individuals who reported consuming alcohol at least once a month, and in 2019 this question included individuals who reported consuming alcohol, including those who consume less than once a month. This change in the questionnaire partly explains the increase in abusive consumption, both in males and in females, since the indicator now includes individuals who engage in abusive consumption regardless of having a monthly consumption of alcoholic beverages. Another limitation refers to the capture of data through the questionnaire, subject to the interviewees’ memory bias.

The high prevalence of alcohol consumption in the Brazilian population, in the pattern of alcohol abuse, weekly consumption or monthly consumption, indicate that this is a political, economic, social, and public health issue in the Brazilian population that deserves attention from the Government bodies and demands public policies. When one considers that the prevalence is increasing, it is clear that the measures taken are still insufficient to contain the advance in the consumption of alcoholic beverages and the damage resulting from this consumption in the Brazilian population. Facing this issue requires the elaboration of public policies aimed at existing national gaps, meeting the new global strategies; it also requires dialogue between the public sphere and civil society and demands intensified monitoring of the measures implemented.
