# Supplementary material for: Consumption of alcoholic beverages in Brazil: estimation of prevalence ratios – 2013 and 2019
Source: Rev Saude Publica. 2023 Mar 29;57:17. doi: 10.11606/s1518-8787.2023057004380 (PMC10118409; doi:10.11606/s1518-8787.2023057004380)
Supplement: Translation

# Translation: Consumo de bebidas alcoólicas no Brasil: estimativa de razões de prevalências – 2013 e 2019

**Authors:** Mariana Gonçalves de Freitas, Sheila Rizzato Stopa, Everton Nunes da Silva

**Affiliations:** I Brasil Universidade de Brasília. Faculdade de Ciências da Saúde. Programa de Pós Graduação em Saúde Coletiva. Brasília, DF, Brasil; II Brasil Ministério da Saúde. Secretaria de Vigilância em Saúde. Departamento de Análise Epidemiológica e Vigilância de Doenças Não transmissíveis. Brasília, DF, Brasil; III Brasil Ministério da Saúde. Secretaria de Ciência, Tecnologia, Inovação e Insumos Estratégicos em Saúde. Departamento de Ciência e Tecnologia. Brasília, DF, Brasil; IV Brasil Universidade de Brasília. Faculdade de Ceilândia. Curso de Saúde Coletiva. Brasília, DF, Brasil

**Keywords:** Consumo de Bebidas Alcoólicas, Fatores de Risco, Fatores Sociodemográficos, Saúde de Gênero

## Abstract

**OBJETIVOS:**

Estimar as prevalências de consumo de bebidas alcoólicas semanal, mensal e abusivo no Brasil em 2013 e 2019, comparar as estimativas do período e estimar a magnitude das diferenças.

**MÉTODOS:**

Análise dos dados do consumo de bebidas alcoólicas na população adulta (18 anos ou mais) da Pesquisa Nacional de Saúde (PNS), 2013 e 2019. O número de entrevistados em 2013 foi de 60.202 e, em 2019, de 88.531. As amostras foram caracterizadas segundo variáveis demográficas, socioeconômicas, de saúde e de consumo de bebidas alcoólicas; e foram comparadas as diferenças de proporções no período, por meio do teste do c2 de Pearson, com aproximação de Rao-Scott e nível de significância de 5%. Foram estimados modelos multivariados de regressão de Poisson para as variáveis de desfecho de consumo mensal, semanal e abusivo de bebidas alcoólicas, com o intuito de estimar a magnitude das diferenças entre as estimativas da PNS 2013 e 2019, por meio da razão de prevalência (RP). Os modelos foram ajustados por sexo e faixa etária e estratificados por sexo e região demográfica.

**RESULTADOS:**

Houve diferença da distribuição da população segundo raça, ocupação, renda, faixa etária, estado civil e escolaridade. Houve aumento do consumo de álcool para todas as variáveis desfecho, com exceção do consumo semanal em homens. A razão de prevalência do consumo semanal foi de 1,02 (IC95% 1,014–1,026), nas mulheres a RP foi de 1,05 (IC95% 1,04–1,06). As maiores razões de prevalência na população geral e por sexo ocorrem para o consumo abusivo. O aumento do consumo semanal por região ocorreu no Sul, Sudeste e Centro-Oeste.

**CONCLUSÕES:**

O homem é o principal consumidor de álcool no Brasil, as razões de prevalência tanto em homens quanto em mulheres demonstram que houve aumento do consumo mensal, semanal e abusivo no período pesquisado, destaca-se que as mulheres têm aumentado o padrão de consumo com maior intensidade do que os homens.

## INTRODUÇÃO

O consumo de bebidas alcoólicas está presente em diversas culturas e regiões do mundo^1^e é influenciado por fatores sociais, psicológicos, comportamentais, econômicos, legais e ambientais^2^. Esses fatores se expressam no padrão de consumo, nas práticas exercidas pela indústria de bebidas alcóolicas e nas políticas públicas de cada país^2^. O volume de álcool puro consumido e o padrão de consumo (quantidade ingerida e frequência) são fatores diretamente relacionados aos danos causados pelo consumo de bebidas alcoólicas^3^. O álcool é um fator relevante de risco para morbimortalidade no Brasil e no mundo e está associado a mais de 200 causas de morte, incluindo cânceres, doenças hepáticas, doenças do aparelho circulatório, acidentes, violências e outras^3^.

Dados da Organização Mundial de Saúde (OMS) apontam que a prevalência do consumo de bebidas alcoólicas tem aumentado nos últimos anos^1^. Em 2016, a prevalência de consumo episódico excessivo de bebidas alcoólicas (cinco ou mais doses em um único episódio, nos últimos 30 dias) entre indivíduos de 15 anos e mais que consomem bebidas alcoólicas foi de 39,5% no mundo e 40,5% na região das Américas^1^. O consumo total de bebidas alcoólicas na população mundial passou de 5,5 litros de álcool puro por habitante em 2005 para 6,4 litros em 2016, podendo chegar a 7 litros em 2025. Nas Américas, o consumo em 2016 foi de 8 litros per capita e no Brasil foi de 7,8 litros (13,4 em homens e 2,4 em mulheres), valores superiores às estimativas globais^1^.

No mundo, o consumo de bebidas alcoólicas está associado a três milhões de mortes e 131,4 milhões de anos de vida ajustados por incapacidades ( *disability-adjusted life years* – DALY)^4^, correspondendo a 5,3% dos óbitos e 5% do total de *DALYs* , em 2016. Considerando a população na faixa etária de 20 a 39 anos, 13,5% das mortes estão associadas ao consumo de bebidas alcoólicasl^1^. Estudo realizado em 30 dos 35 países das Américas, entre 2013 e 2015, apontou que mais de 85 mil mortes (1,4% do total) foram totalmente atribuíveis ao álcool, sendo que 83,1% desses óbitos foram em homens^5^, 64,9% dos óbitos ocorreram em indivíduos com menos de 60 anos de idade^5^. Esses óbitos são evitáveis e estão associados a elevada carga de doenças e mortalidade prematura. Estimativas apontam que o consumo de bebidas alcoólicas contribuiu com mais de 300 mil mortes nas Américas em 2012 (5,5% do total)^3^.

Dados do *Global Burden of Disease* 2017 apontam que 6,2% dos óbitos ocorridos no Brasil estão relacionados ao consumo de bebidas alcoólicas e indica que o álcool é o terceiro fator de risco para carga de doenças no Brasil e o quarto fator em todo o mundo^6^. Além da carga de morbimortalidade, o consumo de bebidas alcoólicas também gera um importante impacto econômico e social na população.

Estudos brasileiros anteriores sobre o consumo de bebidas alcoólicas e as variáveis de associação trazem informações relevantes para compreender o consumo de bebidas alcoólicas no Brasil e quem são os principais consumidores, de acordo com as características demográficas e socioeconômicas^7 , 8^. De modo a ampliar o conhecimento sobre o tema, esta pesquisa acrescenta informações sobre os padrões de consumo de bebidas alcoólicas e, principalmente, sobre a intensidade de aumento do consumo na população brasileira no período estudado. Isso é um ponto relevante para os elaboradores de políticas públicas no dimensionamento e enfrentamento dos problemas decorrentes do uso do álcool. Os objetivos deste estudo foram descrever o perfil da população brasileira, segundo características socioeconômicas e demográficas, em 2013 e 2019; descrever as prevalências de consumo de bebidas alcoólicas semanal, mensal e abusivo por sexo; estimar as prevalências de consumo semanal de bebidas alcoólicas segundo região demográfica; e comparar as estimativas do período e estimar a magnitude das diferenças.

## MÉTODOS

Este trabalho é um estudo transversal dos dados provenientes da Pesquisa Nacional de Saúde (PNS), nas edições 2013 e 2019. A PNS é um inquérito domiciliar, de base populacional, realizado pelo Instituto Brasileiro de Geografia e Estatística (IBGE), em parceria com o Ministério da Saúde. Seus dados, coletados em amostra representativa, produziram um dos mais fidedignos retratos das condições de vida e saúde da população residente no Brasil^9 , 10^.

O processo amostral da PNS se deu por conglomerados em três estágios de seleção. No primeiro estágio, foram estratificadas as Unidades Primárias de Amostragem (UPA), compostas pelos setores censitários, descritos anteriormente, por meio de seleção aleatória simples. No segundo estágio, foram selecionados, aleatoriamente, os domicílios em cada unidade selecionada no primeiro estágio. No terceiro, um morador foi selecionado com equiprobabilidade entre os demais moradores do domicílio. Esse morador tinha, no momento da pesquisa 18 anos ou mais, em 2013, e 15 anos ou mais, em 2019.

O questionário da PNS 2019 foi elaborado com base na edição anterior, com o intuito de manter o máximo de comparação entre as edições, uma vez que a PNS é usada para o monitoramento de indicadores nacionais e internacionais, como os do Plano de Ações Estratégicas para o Enfrentamento das Doenças Crônicas Não Transmissíveis (DCNT) no Brasil 2011-2022^11^e dos Objetivos de Desenvolvimento Sustentável (ODS)^12^.

A população incluída na pesquisa reside permanentemente em domicílios particulares do Brasil, ou seja, setores censitários especiais como, bases militares, penitenciárias, instituições de longa permanência, conventos, hospitais etc. não são incluídos na amostra bem como os setores localizados em terras indígenas.

A amostra de 2013 foi composta por 69.994 domicílios, resultando em 64.348 entrevistas, com taxa de não resposta de 8,1%. Em 2019, foram visitados 108.525 domicílios, dos quais 94.114 entrevistas foram coletadas e a taxa de não resposta foi de 6,4%. Em função de seu processo amostral, faz-se necessário utilizar algoritmo capaz de considerar efeitos de sua estratificação e conglomeração para estimar indicadores. Assim, fatores de ponderação foram calculados pelo inverso da probabilidade de seleção em cada estágio, adicionando um fator de correção para perdas^13^.

Este estudo analisou dados pertinentes à população adulta (18 anos ou mais de idade), proveniente de ambas as edições da PNS, referentes ao consumo de bebidas alcoólicas referido pelos entrevistados. O número de entrevistados com 18 anos de idade ou mais em 2013 foi de 60.202 e, em 2019, de 88.531.

Foram considerados os desfechos de interesse: consumo de bebidas alcoólicas mensal, obtido por meio da pergunta P27 “Com que frequência, o(a) sr(a) costuma consumir bebida alcoólica?”; consumo de bebidas alcoólicas semanal, obtido por meio da pergunta P28 da PNS 2013 “Quantos dias por semana, o(a) sr(a) costuma tomar alguma bebida alcoólica?” e P28a, da PNS 2019, “Quantos dias por semana, o(a) sr(a) costuma consumir alguma bebida alcoólica?”; e consumo abusivo de álcool nos últimos 30 dias, obtido a partir da pergunta P32, da PNS 2013, “Nos últimos 30 dias, o sr. chegou a consumir 5 ou mais doses de bebida alcoólica em uma única ocasião?” (se homem)” ou “Nos últimos 30 dias, a sra chegou a consumir 4 ou mais doses de bebida alcoólica em uma única ocasião? (se mulher)” e a pergunta P32a do questionário da PNS 2019 “Nos últimos 30 dias, o(a) sr(a) chegou a consumir 5 ou mais doses de bebida alcoólica em uma única ocasião?”. Para exemplificar, uma dose bebida alcoólica equivale a: 1 lata de cerveja ou 1 taça de vinho ou 1 dose de cachaça, whisky ou qualquer outra bebida alcoólica destilada. Ressaltamos que houve uma mudança na classificação de consumo abusivo para o sexo feminino adotada pela PNS em 2019, de acordo com orientação da OMS^1^.

Para o cálculo do consumo semanal e do consumo abusivo foi considerado no denominador todos os respondentes do questionário maiores de 18 anos. A partir das perguntas P028 e P029 foi criada a variável *consumodiario,* por meio da multiplicação dessas perguntas e divisão por sete, para estimar a dose diária de álcool consumida. Em seguida, esse valor foi multiplicado por 12, para estimar a dose diária em gramas. Para essa variável foi utilizada a dose padrão de 12 gramas de álcool puro por dose.

As amostras em ambos os anos de estudo foram caracterizadas segundo variáveis demográficas e socioeconômicas: sexo (feminino ou masculino), faixa etária (18 a 29, 30 a 39, 40 a 59, 60 anos e mais), raça/cor (branca, preta e parda), níveis de escolaridade (sem escolaridade até fundamental incompleto, fundamental completo a médio incompleto, médio completo a superior incompleto, superior completo), estado civil (casada(o), divorciada(o) ou desquitada(o), viúva(o) e solteira(o)), ocupação (ocupadas ou desocupadas), renda em salários mínimos (SM) (sem renda, até ½ SM, de ½ SM a 1 SM, de 1 SM até 2 SM, maior que 2 SM), Grandes Regiões (Norte, Nordeste, Sul, Sudeste, Centro-Oeste) e setor censitário (urbana ou rural). A amostra também foi caracterizada por variáveis de saúde e variáveis relacionadas ao consumo de bebidas alcoólicas: autopercepção de saúde (muito boa/boa, regular, ruim/muito ruim), consumo de tabaco (sim, não), consumo de bebidas alcoólicas por dias da semana (0, 1, 2, 3-6, 7), consumo de bebidas alcoólicas em doses (1, 2-4, 5-9, > 10) e consumo de bebidas alcoólicas em gramas por dia (12, 24, 36, 48, 60 e 72).

As análises foram realizadas em um único banco de dados, onde as variáveis supracitadas foram agrupadas para possibilitar a comparação entre as duas edições da PNS. A comparação se deu por meio da estimação das proporções e seus intervalos de 95% de confiança. Com o objetivo de verificar possíveis mudanças entre os dados das duas edições da PNS, foi utilizado o teste do c2 de Pearson, com aproximação de Rao-Scott e nível de significância de 5%. Considerando as variáveis de desfecho consumo mensal de bebidas alcoólicas, consumo semanal de bebidas alcoólicas e consumo abusivo de bebidas alcoólicas foram estimados modelos multivariados de regressão de Poisson, com o intuito de estimar a magnitude das diferenças entre as estimativas das PNS 2013 e 2019, através da razão de prevalência. Os modelos foram ajustados por sexo e faixa etária. Os modelos que foram estratificados por sexo, foram ajustados por faixa etária. As análises foram realizadas no software Stata 14.0, com utilização do módulo *survey* , que considera os efeitos da amostragem complexa na estimação dos parâmetros.

Em ambas as edições, a PNS obteve aprovação da Comissão Nacional de Ética em Pesquisa (CONEP), sob os pareceres n. 328.159, de 26 de junho de 2013, e n. 3.529.376, de 23 de agosto de 2019. Os dados da PNS são públicos e estão disponíveis em https://www.pns.icict.fiocruz.br/bases-de-dados/^14^.

## RESULTADOS

A Tabela 1 apresenta o perfil da população brasileira segundo características socioeconômicas e demográficas em 2013 e em 2019. Observamos um aumento do autorrelato de pessoas de cor preta e uma diminuição das pessoas de cor branca entre 2013 e 2019 (p < 0,001); aumento da proporção da população idosa (60 anos e mais), passando de 18,06% para 21,61%, e uma diminuição da população mais jovem de 18 a 29 anos, passando de 26,11% para 22,10% (p < 0,001); aumento da proporção de divorciados ou desquitados judicialmente, passando de 6,41% para 7,08%; aumento da proporção de indivíduos com nível superior (de 12,72% para 15,83%) e redução da população sem escolaridade e com fundamental incompleto (de 39,98% para 34,76%) (p < 0,001); redução da população ocupada, passando de 94,72% para 92,78% (p < 0.001); aumento da proporção de pessoas que recebem meio salário-mínimo, dos 19,40% em 2013 para 21,27% em 2019, e houve redução de pessoas que recebem mais de dois salários-mínimos, passando de 21,47% para 20,62% (p = 0,001). Não houve diferença estatística entre os anos em relação às proporções de sexo, região demográfica e setor censitário.

**Tabela 1:** Distribuição percentual da população brasileira de 18 anos ou mais, segundo características demográficas e socioeconômicas e comparação das diferenças no período, Brasil 2013 e 2019.

	2013		2019		p^a^
	
	%	IC95%	%	IC95%	
Sexo					
Feminino	52,90	52,13–53,66	53,16	52,56–53,76	
Masculino	47,10	46,34–47,87	46,84	46,24–47,44	0,5922
Faixa etária					
60 anos e mais	18,06	17,48–18,65	21,61	21,08–22,16	< 0,001
40 a 59 anos	34,24	33,59–34,91	35,30	34,71–35,89	
30 a 39 anos	21,59	21,0–22,19	20,99	20,48–21,51	
18 a 29 anos	26,11	25,48–26,76	22,10	21,51–22,70	
Raça					
Branca	48,21	47,39–49,03	43,91	43,18–44,64	< 0,001
Preta	9,26	8,81–9,73	11,64	11,23–12,06	
Parda	42,53	41,75–43,31	44,45	43,77–45,14	
Escolaridade					
Sem escolaridade a fundamental incompleto	38,98	38,12–39,84	34,76	34,1–35,42	< 0,001
Fundamental completo a Médio incompleto	15,52	14,98–16,07	14,48	14,06–14,91	
Médio completo a Superior incompleto	32,79	32,08–33,5	34,94	34,33–35,55	
Superior completo	12,72	12,02–13,45	15,83	15,19–16,48	
Estado civil					
Casado (a)	44,46	43,67–45,25	43,88	43,2–44,55	0,0285
Divorciado (a) ou desquitado (a) judicialmente	6,41	6,09–6,76	7,08	6,82–7,36	
Viúvo (a)	6,66	6,36–6,98	6,85	6,58–7,14	
Solteiro (a)	42,47	41,73–43,22	42,19	41,52–42,85	
Ocupação					
Ocupadas	94,72	94,28–95,13	92,08	91,62–92,52	< 0,001
Desocupadas	5,28	4,87–5,72	7,92	7,48–8,38	
Renda					
Sem renda	1,02	0,89–1,17	0,89	0,80–0,98	0,0011
1/2 salário	19,40	18,81–20,00	21,27	20,72–21,82	
1 salário	29,32	28,57–30,08	29,08	28,47–29,69	
2 salários	28,79	28,11–29,49	28,16	27,55–28,77	
> 2 salários	21,47	20,61–22,36	20,62	19,89–21,36	
Região demográfica					
Norte	7,47	7,25–7,70	7,85	7,59–8,12	0,3862
Nordeste	26,46	25,92–27,01	26,45	25,92–27,00	
Sudeste	43,91	43,22–44,61	43,44	42,65–44,24	
Sul	14,75	14,33–15,18	14,68	14,27–15,11	
Centro-Oeste	7,41	7,21–7,62	7,57	7,293–7,854	
Setor censitário					
Rural	13,83	13,37- 14,30	13,82	13,44–14,22	0,9900
Urbano	86,17	85,70–86,63	86,18	85,78–86,56	

Fonte: Pesquisa Nacional de Saúde, 2013 e 2019

IC95%: intervalo de confiança de 95%.

^a^c2 de Pearson, com aproximação de Rao-Scott.

A Tabela 2 apresenta variáveis relacionadas à saúde e ao consumo de bebidas alcoólicas. Não houve diferença estatística entre os anos em relação à autopercepção de saúde. Houve redução da prevalência de fumantes no período, passando de 14,65% para 12,59%, com p valor < 0,001. As variáveis relacionadas ao consumo de bebidas alcoólicas apresentaram alteração nas proporções: número de dias da semana que a pessoa consome álcool, número de doses consumidas e consumo diário de álcool em gramas. Observa-se que houve aumento do consumo para essas três variáveis entre 2013 e 2019 (p < 0,001). Em 2013, 13,55% da população referiu consumir em média uma dose de álcool por dia, em 2019 essa proporção foi para 14,70%. Dos entrevistados, 5,41% referiram consumir 24 gramas de álcool por dia em 2013, passando para 6,06% em 2019. Em 2013, 2,40% referiram consumir 36 gramas de álcool por dia, já em 2019 o percentual subiu para 2,83% (p < 0,001).

**Tabela 2:** Distribuição percentual da população brasileira de 18 anos ou mais, segundo características de saúde e de consumo de bebidas alcoólicas e comparação das diferenças no período, Brasil 2013 e 2019.

	2013		2019		p^a^
	
	%	IC95%	%	IC95%	
Autopercepção de saúde					
Muito boa/Boa	66,19	65,48–66,90	66,11	65,5–66,72	0,9752
Regular	28,01	27,37–28,67	28,10	27,57–28,64	
Ruim/Muito ruim	5,79	5,49–6,11	5,79	5,53–6,05	
Fumante					
Não	85,35	84,85–85,84	87,41	87,01–87,81	< 0,001
Sim	14,65	14,16–15,15	12,59	12,19–12,99	
Número de dias da semana					
0	9,72	8,91–10,59	12,09	11,39–12,83	0,0001
1	40,91	39,51–42,33	39,28	38,20–40,36	
2	27,09	25,80–28,42	26,95	25,98–27,94	
3 a 6	15,20	14,25–16,21	15,76	14,94–16,61	
7	7,08	6,40–7,83	5,93	5,41–6,49	
Número de doses					
1	18,86	17,64–20,14	21,13	20,32–21,97	< 0,001
2 a 4	44,30	42,84–45,77	45,85	44,81–46,90	
5 a 9	22,48	21,32–23,69	21,21	20,41–22,04	
>10	14,36	13,48–15,28	11,81	11,23–12,40	
Consumo de bebidas alcoólicas em gramas por dia					
0	76,08	75,38–76,77	73,61	73,03–74,19	< 0,001
12	13,55	13,00–14,11	14,70	14,25–15,16	
24	5,41	5,08–5,77	6,06	5,79–6,34	
36	2,40	2,18–2,64	2,83	2,58–3,11	
48	0,84	0,71–0,99	1,05	0,93–1,18	
60	0,49	0,42–0,58	0,57	0,49–0,66	
72	1,24	1,11–1,38	1,18	1,05–1,33	

Fonte: Pesquisa Nacional de Saúde, 2013 e 2019

IC95%: intervalo de confiança de 95%.

^a^c2 de Pearson, com aproximação de Rao-Scott.

A Tabela 3 apresenta as prevalências mensal, semanal e de consumo abusivo de bebidas alcoólicas em 2013 e em 2019. Para todas as variáveis de consumo de bebidas alcoólicas houve aumento do consumo com diferença estatística entre os anos, com exceção do consumo semanal em homens.

**Tabela 3:** Prevalências e razões de prevalências ajustadas do consumo mensal, semanal e abusivo de bebidas alcoólicas na população brasileira a partir de 18 anos, Brasil, 2013 e 2019.

	2013		2019		RP^a^	IC95%	p^b^

	%	IC95%	%	IC95%			
Consumo de bebidas alcoólicas mensal	26,49	25,76–27,24	30,02	29,41–30,64	1,009	1,007–1,011	< 0,001
Consumo de bebidas alcoólicas mensal–sexo masculino	39,23	38,14–40, 34	41,16	40,28–42,05	1,004	1,002–1,007	0,001
Consumo de bebidas alcoólicas mensal–sexo feminino	15,14	14,38–15,94	20,20	19,48–20,94	1,014	1,011- 1,017	< 0,001
Consumo de bebidas alcoólicas semanal	23,92	23,23–24,62	26,39	25,81–26,97	1,020	1,014–1,026	< 0,001
Consumo de bebidas alcoólicas semanal–sexo masculino	36,27	35,20–37,34	37,09	36,20–37,99	1,006	0,999–1,012	0,078
Consumo de bebidas alcoólicas semanal–sexo feminino	12,92	12,23–13,65	16,96	16,29–17,64	1,053	1,041–1,065	< 0,001
Consumo abusivo de álcool	13,62	13,11–14,15	17,06	16,59–17,55	1,045	1,038–1,053	< 0,001
Consumo abusivo de álcool–sexo masculino	21,52	20,62–22,45	26,00	25,17–26,84	1,037	1,028–1,046	< 0,001
Consumo abusivo de álcool–sexo feminino	6,59	6,14–7,07	9,20	8,74–9,67	1,069	1,052–1,083	< 0,001

Fonte: Pesquisa Nacional de Saúde, 2013 e 2019

IC95%: intervalo de confiança de 95%.

^a^RP ajustada por sexo e idade.

^b^c2 de Pearson, com aproximação de Rao-Scott

Os dados da razão de prevalência (RP) de 2019 em relação a 2013 mostram que, na população geral, houve aumento do consumo mensal de bebidas alcoólicas no período (RP de 1,009 IC95% 1,007–1,011). O consumo de bebidas alcoólicas mensal no sexo masculino teve razão de prevalência de 1,004 (IC95% 1,002–1,007) e no sexo feminino a RP foi de 1,014 (IC95% 1,011–1,017), demonstrando que o maior aumento do consumo ocorreu nas mulheres ( tabela 3 ).

Os dados de consumo semanal de bebidas alcoólicas também revelam aumento do consumo no período (RP 1,02 IC95% 1,014–1,026), nas mulheres a RP foi de 1,05 (IC95% 1,04–1,06). As maiores razões de prevalência na população geral e por sexo estão relacionadas ao consumo abusivo de bebidas alcoólicas. Observa-se que o homem é o principal consumidor de álcool no Brasil, as razões de prevalência tanto em homens quanto em mulheres demonstram que houve aumento do consumo no período, tanto o consumo mensal quanto o semanal e o abusivo, destaca-se que houve elevação no padrão de consumo com maior intensidade entre as mulheres em relação aos homens ( Tabela 3 ).

Observa-se que as prevalências de consumo semanal de bebidas alcoólicas no Brasil se diferenciam entre as regiões demográficas ( Tabela 4 ). Em 2013, a região de maior consumo foi a Sul, seguida da região Centro-Oeste, Sudeste, Nordeste e Norte. Essa ordem se altera em 2019, quando a região Sudeste passa a ocupar o segundo lugar em termos de prevalência de consumo semanal de bebidas alcoólicas no Brasil e região Centro-Oeste ocupa o terceiro lugar. A região com maior aumento do consumo semanal de álcool no país foi a Sudeste (RP=1,038 IC95% 1,028–1,049), seguida da região Sul (RP=1,22 IC95% 1,008–1,036) e região Centro-Oeste(RP = 1,016; IC95% 1,004 a 1,029). Nas regiões Norte e Nordeste não houve diferença estatística para razão de prevalência.

**Tabela 4:** Prevalências e razões de prevalências ajustadas do consumo semanal de bebidas alcoólicas na população brasileira a partir de 18 anos, segundo região demográfica do Brasil, 2013 e 2019.

	2013		2019		RP^a^	IC95%	p^b^
	
	%	IC95%	%	IC95%			
Norte	18,80	17,40–20,29	17,21	16,29–18,17	0,991	0,976–1,006	0,239
Nordeste	22,28	21,26–23,34	20,72	19,96–21,50	0,993	0,984–1,002	0,148
Sudeste	24,09	22,85–25,38	29,71	28,62–30,83	1,038	1,028–1,049	< 0,001
Sul	28,18	26,33–30,12	31,21	29,79–32,68	1,022	1,008–1,036	0,001
Centro-Oeste	25,38	23,89–26,94	27,28	25,97–28,64	1,016	1,004–1,029	0,011

Fonte: Pesquisa Nacional de Saúde, 2013 e 2019

IC95%: intervalo de confiança de 95%.

^a^RP ajustada por sexo e idade.

^b^c2 de Pearson, com aproximação de Rao-Scott.

## DISCUSSÃO

As razões de prevalência do período demonstram que houve aumento do consumo mensal, semanal e abusivo de bebidas alcoólicas entre 2013 e 2019, o que também foi observado na análise estratificada por sexo. Para todos os indicadores de padrão de consumo de bebidas alcoólicas trabalhados nesse estudo, o aumento da prevalência mensurado pela razão de prevalência no período foi maior no sexo feminino comparado ao sexo masculino. Apesar do consumo de bebidas alcoólicas em mulheres ter um aumento mais expressivo, os homens permanecem os principais consumidores de bebidas alcoólicas, o que está alinhado com as estimativas internacionais^1^.

O aumento do consumo semanal de bebidas alcoólicas também foi observado nas regiões Sudeste, Sul e Centro-Oeste do Brasil, que já apresentavam as maiores prevalências de consumo. O menor consumo ocorreu no Norte e Nordeste. Dados da OMS apontam que as maiores prevalências de consumo de bebidas alcoólicas ocorrem em países de maior renda^1^. Isso pode ajudar a compreender por que nas regiões Norte e Nordeste foram observadas as menores prevalências. Considerando as prevalências de consumo abusivo nas capitais, as maiores prevalências ocorrem em Salvador e Florianópolis, enquanto o menor consumo de álcool ocorre em Manaus e Fortaleza. O consumo abusivo é maior entre homens de 25 a 34 anos e de alta escolaridade^15^.

Estudo com dados da PNS 2013 aponta que o consumo recente (nos últimos 30 dias) de álcool naquele ano foi de 26,5%^7^. No nosso estudo, esses resultados são correspondentes ao consumo mensal de álcool. O consumo recente de álcool entre homens foi associado com cor de pele branca, faixa etária mais jovem, solteiro e residente em área urbana. Já em mulheres, esse consumo se associou com faixa etária mais jovem, aumento da escolaridade, solteira ou separada e residente de área urbana. Esses dados agregam informações ao nosso estudo, já que seu objetivo principal foi investigar as diferenças do perfil da população entre 2013 e 2019 bem como a evolução das prevalências de consumo de bebidas alcoólicas no período.

Pesquisa que investigou o consumo pesado de bebidas alcoólicas no Brasil a partir de dados das PNS 2013 e 2019 identificou que 6,1% dos brasileiros tinham padrão de consumo pesado em 2013, passando para 7,3% em 2019. As maiores prevalências foram em homens, jovens e de baixa escolaridade, solteiro e residentes em área urbana. Os resultados supracitados diferem dos dados de consumo abusivo episódico desta análise porque foi utilizada a referência de beber pesado do *Center for Disease Control and Prevention* (CDC), que é definido pela ingestão de 8 ou mais doses de bebida alcoólica por semana para as mulheres e 15 ou mais doses para homens^16^. O padrão referido pelo CDC implica em maior número de doses e considera o consumo ao longo da semana, no entanto, neste estudo foi considerado o consumo abusivo em um mesmo episódio.

O consumo episódico pesado aumenta o risco de ocorrência dos acidentes e violências, bem como o desenvolvimento de doenças relacionadas ao álcool. Revisão da literatura apontou a necessidade de estudos adicionais que investiguem o papel da relação entre o padrão de consumo adotado e o volume médio de álcool consumido, de modo a obter estimativas de risco mais precisas e compreender melhor a natureza das relações álcool-doença^17^.

O aumento do consumo de bebidas alcoólicas na população brasileira acompanha a tendência de aumento das Américas prevista pela OMS^1^. Essa intensificação do consumo é um alerta para os elaboradores de políticas públicas. Vários estudos atualmente revelam que não existe consumo de bebidas alcoólicas que não ofereça riscos à saúde^18 , 19^. Esses estudos, inclusive, afirmam que os possíveis efeitos protetores cardiovasculares são inferiores aos danos relacionados ao consumo de bebidas alcoólicas^19^. O consumo semanal de álcool foi associado positivamente à mortalidade prematura e às principais causas de mortalidade excessiva foram cânceres, doenças vasculares e causas externas^20^. Considerando o aumento do consumo abusivo de álcool, cabe destacar que o risco para mortalidade por todas as causas aumenta com o aumento da quantidade de álcool ingerida^19 , 20^.

Durante a pandemia de covid-19 houve mudança de padrão do consumo de bebidas alcoólicas, associada ao isolamento social, à faixa etária e à saúde mental^21^. Pesquisa que avaliou a associação entre o aumento do consumo de bebidas alcoólicas e saúde mental identificou RP de 1,64 (IC95% 1,21–2,23) entre indivíduos com sintomas depressivos comparados à indivíduos sem esses sintomas e RP de 1,41 (IC95% 1,20–1,66) em pessoas com sintomas de ansiedade^21^. A associação entre álcool e saúde mental foi mais expressiva entre pessoas acima de 60 anos^21^. Dados de estudo transversal demonstram que 17,6% da população brasileira relatou aumento do consumo de bebidas alcoólicas durante a pandemia, entre indivíduos de 30 a 39 anos esse aumento foi de 24,6%. Não houve diferença do aumento de consumo entre os sexos^22^. Serão necessários novos estudos para saber se esse aumento pode representar um novo padrão de consumo, o que se poderá identificar em edições futuras da PNS.

Considerando as políticas e diretrizes que norteiam as ações dos países no enfrentamento às questões decorrentes do consumo de bebidas alcoólicas, destaca-se a *Estratégia global para reduzir o uso nocivo do álcool* ^23^, que define ações que regulamentam a comercialização do álcool, a disponibilidade, a taxação, dentre outras medidas. A OMS lançou em 2018, o pacote técnico *Safer* que representa um fortalecimento e atualização da *Estratégia Global* e inclui cinco áreas de intervenção nacional e subnacional^24^. O Safer é uma ferramenta que auxilia os países a alcançar as metas definidas nos Objetivos de Desenvolvimento Sustentáveis^25^. Os ODS têm dentre suas metas o monitoramento do consumo de álcool em litros de álcool puro per capita em indivíduos de 15 anos e mais^25^.

No Brasil, o *Plano de Ações Estratégicas para o Enfrentamento de Doenças Crônicas não transmissíveis 2011-2022* definiu como metas nacionais a redução da prevalência do consumo abusivo de álcool em 10%, e como estratégias o aumento dos impostos sobre o álcool, medidas de fiscalização da venda de bebidas alcoólicas para menores de 18 anos, controle dos pontos de vendas e medidas educativas^11^. Em 2021, foi publicada a nova edição do plano, para o período de 2021 a 2030, que trouxe o balanço da primeira década. Os dados Vigitel 2019 indicam que o consumo abusivo no Brasil foi de 18,8%^26^. A meta de redução do consumo abusivo do plano de ações era atingir a prevalência de 16,3% em 2022. Desse modo, as projeções do Ministério da Saúde indicam que o Brasil não atingirá essa meta dentro do período do primeiro plano^27^. Sendo assim, a meta foi renovada no plano 2021–2030. O aumento do preço e diminuição da disponibilidade são custo-efetivas e estão entre as melhores práticas definidas^23 , 28^. A tributação do álcool também é um mecanismo eficaz, principalmente entre adolescentes e indivíduos que praticam consumo pesado de bebidas alcoólicas^29^.

No Brasil, a Aliança para o Controle do Tabaco (ACT) realiza trabalho de *advocacy* com legisladores para intensificar a pauta de regulamentação do álcool. O Ministério Público de São Paulo é responsável pela campanha: “Cerveja também é álcool”^30^, que propõe a alteração do artigo 1º da Lei Federal 9.294/96^31^, a qual permite a propaganda de cerveja, pois a bebida possui graduação alcoólica inferior a 0,5 grau Gay-Lussac. As campanhas publicitárias de álcool exercem forte influência no público que ainda não consome, dentre eles os adolescentes, um grupo de potenciais consumidores de interesse da indústria^2 , 29^. A indústria de bebidas alcóolicas ocupa um papel relevante no cenário nacional e internacional relacionado ao consumo, uma vez que influencia na formulação de políticas, fazendo o gerenciamento dos interesses comerciais das indústrias no espaço de decisões políticas^32 , 33^.

## CONCLUSÃO

Uma limitação deste estudo é a mudança do indicador de consumo abusivo de álcool na PNS 2019 comparado à forma de captação desse dado em 2013. Em 2019, a pesquisa considerou como consumo abusivo de álcool a ingestão de 5 doses ou mais de álcool em uma mesma ocasião para homens e mulheres^10^. Em 2013, a pesquisa considerou consumo abusivo 4 doses para mulheres e 5 doses para homens em uma única ocasião^9^. Desse modo, o aumento do consumo abusivo de bebidas alcoólicas em mulheres entre 2013 e 2019 poderia ser ainda mais expressivo, já que a alteração do indicador mencionado implicou em aumento do número de doses ingeridas em mulheres. Essa mudança partiu da equipe do Ministério da Saúde para alinhar às recomendações da OMS^1^. Além disso, em 2013 essa pergunta sobre consumo abusivo de álcool era respondida por indivíduos que informaram consumir álcool pelo menos uma vez por mês, em 2019, essa pergunta incluiu indivíduos que informaram consumir álcool, incluindo aqueles que consomem menos de uma vez por mês. Essa alteração no questionário explica em parte o aumento do consumo abusivo, tanto em homens quanto em mulheres, visto que o indicador passou a incluir indivíduos que realizam o consumo abusivo independente de apresentarem um consumo mensal de bebidas alcoólicas. Outra limitação refere-se à captação dos dados por meio do questionário, sujeito a viés de memória do respondente.

As elevadas prevalências de consumo de bebidas alcoólicas na população brasileira, seja no padrão de consumo abusivo de álcool, consumo semanal ou consumo mensal apontam que se trata se um problema político, econômico, social e de saúde pública na população brasileira que merece atenção dos órgãos governamentais e demanda políticas públicas. Quando se considera ainda que as prevalências estão aumentando, fica claro que as medidas tomadas são insuficientes ainda para conter o avanço no consumo de bebidas alcoólicas e os danos decorrentes desse consumo na população brasileira. O enfrentamento desse problema exige elaboração de políticas públicas voltadas para lacunas nacionais existentes, atendendo às novas estratégias globais; requer também diálogo entre a esfera pública e a sociedade civil; e demanda intensificação da fiscalização das medidas implementadas.
